# Effects of Co-Worker and Supervisor Support on Job Stress and Presenteeism in an Aging Workforce: A Structural Equation Modelling Approach

**DOI:** 10.3390/ijerph13010072

**Published:** 2015-12-23

**Authors:** Tianan Yang, Yu-Ming Shen, Mingjing Zhu, Yuanling Liu, Jianwei Deng, Qian Chen, Lai-Chu See

**Affiliations:** 1 Department of Organization and Human Resource Management, School of Management and Economics, Beijing Institute of Technology, Beijing 100081, China; teddy.tianan.yang@gmail.com; 2 Institute of Medical Informatics, Biometry and Epidemiology (IBE), Pettenkofer School of Public Health, Faculty of Medicine Ludwig-Maximilians University Munich, Munich 81377, Germany; shen@ibe.med.uni-muenchen.de; 3 Institute of Psychology, Chinese Academy of Sciences, Beijing 100101, China; zhumj@psych.ac.cn; 4 Human Resources Department, Guangdong Women and Children Hospital, Guangzhou 511442, China; liuyuanling60@126.com; 5 Department of Public Administration, School of Management and Economics, Beijing Institute of Technology, Beijing 100081, China; 111605@bit.edu.cn; 6 Medical Affair Department, Peking Union Medical College Hospital, Chinese Academy of Medical Sciences, Beijing 100032, China; chqhust@163.com; 7 Department of Public Health, Medical College, Chang Gung University, Taoyuan 33302, Taiwan; 8 Biostatistics Core Laboratory, Molecular Medicine Research Center, Chang Gung University, Taoyuan 33302, Taiwan

**Keywords:** co-worker support, supervisor support, job stress, presenteeism, structural equation modelling

## Abstract

We examined the effects of co-worker and supervisor support on job stress and presenteeism in an aging workforce. Structural equation modelling was used to evaluate data from the 2010 wave of the Health and Retirement Survey in the United States (*n* = 1649). The level of presenteeism was low and the level of job stress was moderate among aging US workers. SEM revealed that co-worker support and supervisor support were strongly correlated (β = 0.67; *p* < 0.001). Job stress had a significant direct positive effect on presenteeism (β = 0.30; *p* < 0.001). Co-worker support had a significant direct negative effect on job stress (β = −0.10; *p* < 0.001) and presenteeism (β = −0.11; *p* < 0.001). Supervisor support had a significant direct negative effect on job stress (β = −0.40; *p* < 0.001) but not presenteeism. The findings suggest that presenteeism is reduced by increased respect and concern for employee stress at the workplace, by necessary support at work from colleagues and employers, and by the presence of comfortable interpersonal relationships among colleagues and between employers and employees.

## 1. Introduction

Presenteeism can be viewed from two distinct perspectives. It is, of course, the literal antonym of absenteeism, and UK and European researchers in management, epidemiology, and occupational health [1,2,3] have investigated this positive aspect of the term, *i.e.*, when employees remain at work, even when they are sick, or overstate their attendance, because of job insecurity due to downsizing and restructuring forces. However, presenteeism can be regarded as an indicator of lost work productivity, and US medical researchers and consultants [4] are concerned about the adverse effects that sickness and specific medical conditions might have on work productivity in organizations. 

In this article, we discuss presenteeism from the perspective of lost work productivity, because such analysis can address gray gaps between the absence of productivity and full productivity [5] and between healthy and unhealthy persons [6]. Moreover, previous studies suggest that further research is needed in order to better understand the potential differences between absenteeism and presenteeism [7,8,9,10,11].

Presenteeism was initially defined in the field of occupational health as the act of attending work while unable to perform effectively due to health problems [12,13]. The increasing attentions has been attracted is because presenteeism is responsible for 3 times and 1.8 times the financial burden of medical illness and absenteeism, respectively [14]. However, with the development of definition of presenteeism, more recently the definition has been extended to include other conditions and events that limit productivity, as suggested by Johns [15]. Advances in public health, medicine, science, and technology have greatly improved the health and life expectancy in developed and developing countries [16,17]. However, although retirement age is now older [18,19], physical and cognitive capabilities nevertheless deteriorate with age. This could lead to productivity loss and increased employer concerns regarding organizational competitiveness. Unfortunately, the extent of presenteeism among aging workers is not well understood.

Employees who have demanding jobs, low decision latitude, job strain, and/or low social support tend to have more sickness absences [20] and, consequently, severe presenteeism [21,22,23]. Job stress occurs when an individual ability cannot meet job demands [24,25]. The job demand–resources model (JD-R) holds that when job demands are high and there are few job resources, employees may suffer more job stressors, which results in high job stress and other consequences. Because job demands must be satisfied if a worker is to remain employed, employees pretend to work hard at the workplace and forgo absences, even while they are sick or not fully productive. The greater the job requirements, the more efforts employees make in meeting them and the greater the probability that they will work while sick, to ensure full-time presence [26].

Fortunately, strong support from co-workers and supervisors improves work environments by relieving employee stress [27,28], which enhances job satisfaction and performance [29] and subsequently reduces presenteeism in enterprises and organizations [30,31]. Supervisors are in positions that can address employee complaints and help employees obtain necessary resources [32]. Co-workers can successfully finish work tasks and reduce stress and presenteeism [33]. In agreement with the buffering model of social support, Cummins [34] reported that employees that had good relationships with supervisors and co-workers are usually successful and productive at work, even when job stress is severe. Although co-worker support and supervisor support are both important in reducing job stress [35], most studies have investigated these two support mechanisms separately [36]. In addition, the relationship between co-worker and supervisor support has rarely been studied. Moreover, the level of co-worker and supervisor support that employees in an aging workforce receive is unclear.

**Figure 1:**
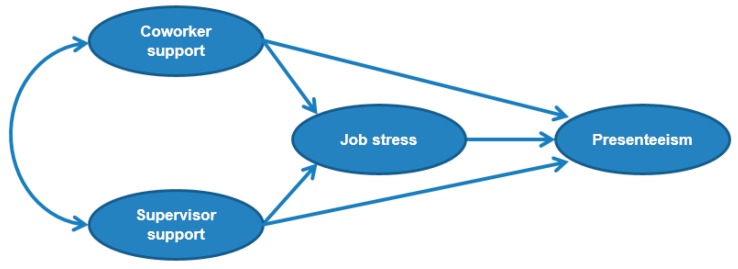
Initial model of how co-worker and supervisor support affect job stress and presenteeism.

Most previous studies [27,34] used linear regression to investigate relationships between co-worker support, supervisor support, job stress, and presenteeism; however, such analysis cannot account for the complicated relationships among these variables. In this study, we used structural equation modelling (SEM) to examine the complicated effects of co-worker and supervisor support on job stress and presenteeism in an aging workforce. We hypothesized that there would be a direct buffer effect of supervisor support on job stress and presenteeism, a direct buffer effect of co-worker support on job stress and presenteeism, and a direct positive effect of job stress on presenteeism (Figure 1).

## 2. Method

### 2.1. Data Source

We conducted a secondary analysis of data from the 2010 wave of the Health and Retirement Survey (HRS) in the United States. The HRS measures the health, retirement, psychosocial factors, and productivity of the labour market and is funded by the National Institute of Aging and the Social Security Administration of the United States. The HRS was initiated in 1992 and recruited persons older than 50 years for participation in biennial surveys based on a multistage area probability sample. To avoid the aging problem and a decrease in the number of participants over time, new samples were added every 6 years [37,38]. In 2006, the HRS added a participant lifestyle questionnaire (PLQ) to their core biennial survey, for a random 50% of the core panel participants. The PLQ was developed by the HRS Psychosocial Working Group and includes a perceived ability to work scale (PAWS), job stress scale, co-worker support scale, and supervisor support scale [39]. Detailed information on the study population and survey methodology has been published elsewhere [40].

In the 2010 wave of the HRS, PLQ data were available for 8080 participants, because only respondents in 2010 who had completed the face-to-face PLQ interview in 2006 again rotated to this mode of data collection [37,38]. Among these 8080 participants, 2730 (33.8%) were still employed and older than 50 years. Among those still employed, 1649 (60.4%) answered at least one question on the “PLQ 2010”, and the percentage of those with missing data was less than 2.6%. Because presenteeism is only observed among employed persons, data from these 1649 participants were analyzed in this study (Table 1).

**Table 1:** Demographic characteristics of the total sample of aging workers and the subset with data from the participant lifestyle questionnaire (this study) in the 2010 wave of the Health and Retirement Survey, USA.

Characteristics	Total Sample (*n* = 2730)		This Study (*n* = 1649)		*p* Value
Sex					0.8819
Male	1237	(45.3%)	750	(45.5%)	
Female	1493	(54.7%)	899	(54.5%)	
Age (years)					<0.0001
50–59	1520	(55.7%)	990	(60.0%)	
60–69	864	(31.6%)	499	(30.3%)	
70–79	309	(11.3%)	150	(9.1%)	
≥80	37	(1.4%)	10	(0.6%)	
Education					0.5158
No degree	244	(8.9%)	151	(9.2%)	
Grade 1–9	133	(4.9%)	87	(5.3%)	
High school diploma	1267	(46.4%)	764	(46.3%)	
Two-year college degree	198	(7.3%)	130	(7.9%)	
Four-year college degree	490	(17.9%)	290	(17.6%)	
Master’s degree	298	(10.9%)	182	(11.0%)	
Professional degree (Ph.D., M.D., J.D.)	87	(3.2%)	37	(2.2%)	
Degree unknown/some college	13	(0.5%)	8	(0.5%)	
Race					0.0277
White	645	(23.6%)	440	(26.7%)	
African American	269	(9.9%)	163	(9.9%)	
Unspecified	114	(4.2%)	63	(3.8%)	
Missing	1702	(62.3%)	983	(59.6%)	
2010 marital status					0.6963
Married	1859	(68.1%)	1115	(67.6%)	
Separated/divorced	527	(19.3%)	326	(19.8%)	
Widowed	176	(6.4%)	111	(6.7%)	
Never married	165	(6.0%)	97	(5.9%)	
Unknown	3	(0.1%)	0	(0.0%)	
Self-rated health					0.9344
Excellent	420	(15.4%)	250	(15.2%)	
Very good	1076	(39.4%)	642	(38.9%)	
Good	831	(30.4%)	513	(31.1%)	
Fair	342	(12.5%)	204	(12.4%)	
Poor	61	(2.2%)	40	(2.4%)	
Chronic diseases					0.7507
Hypertension	1312	(48.1%)	805	(48.8%)	
Diabetes	471	(17.3%)	304	(18.4%)	
Cancer (excluding skin cancer)	236	(8.6%)	137	(8.3%)	
Lung disease	137	(5.1%)	79	(4.8%)	
Heart disease	368	(13.5%)	224	(13.6%)	
Emotional/psychiatric problems	326	(11.9%)	210	(12.8%)	
Arthritis	1163	(42.6%)	682	(41.4%)	
Working hours per week					<0.0001
<10	89	(3.3%)	35	(2.1%)	
10–19	181	(6.6%)	80	(4.9%)	
20–29	287	(10.5%)	146	(8.9%)	
30–39	458	(16.8%)	277	(16.8%)	
40–49	1165	(42.7%)	808	(49.0%)	
≥50	440	(16.1%)	262	(15.9%)	
Unspecified	30	(1.1%)	8	(0.5%)	
Missing data	80	(2.9%)	33	(2.0%)	

### 2.2. Variables and Instruments

*Presenteeism* was measured using PAWS because that instrument has been validated as a robust indicator of perceived productivity loss [41]. PAWS is a reliable and valid instrument and has acceptable psychometric properties [42]. The Cronbach α for PAWS was 0.89 [39] for both the HRS Psychosocial Working Group and the present study. The PAWS consists of four items, e.g., “How many points would you give your current ability to work?” (Table 2). Each item is rated from 0 (cannot currently work at all) to 10 (work ability is currently at its lifetime best). To have scores reflect the magnitude of presenteeism, we changed score directionality by subtracting the original PAWS scores from 10. Hence, higher values for the new presenteeism score represent greater presenteeism.

*Job stress* was measured by using the six items of the “job stress scale” [39,43], e.g., “I am under constant time pressure due to a heavy workload” (Table 2). Each item was rated on a four-point scale (1 = strongly disagree, 2 = disagree, 3 = agree, 4 = strongly agree). Higher values reflect greater job stress. The Cronbach α for this scale was 0.72 for the HRS Psychosocial Working Group [39] and 0.73 for the present study. This instrument has acceptable psychometric properties [43].

*Work support* from respondents’ colleagues and supervisors was measured using the three-item “co-worker support scale” [39] and four-item “supervisor support scale” [39], respectively, e.g., “my co-workers help me in crisis situations at work” and “my supervisor takes pride in my accomplishments at work”, respectively (Table 2). Each item was rated on a four-point scale (1 = strongly disagree, 2 = disagree, 3 = agree, 4 = strongly agree). Higher values reflect greater support. The Cronbach α values for these scales were 0.90 for co-worker support and 0.92 for supervisor support, for the HRS Psychosocial Working Group [39], and 0.89 and 0.92, respectively, for our study. These two instruments have acceptable psychometric properties [44,45].

**Table 2:** Means (SD) for presenteeism (P), job stress, co-worker support (CS), and supervisor support (SS) items.

Variables	Items	Mean	SD
Presenteeism (0–10)	P1: How many points would you give your current ability to work?	1.38	1.46
	P2: Thinking about the physical demands of your job, how do you rate your current ability to meet those demands?	1.31	1.52
	P3: Thinking about the mental demands of your job, how do you rate your current ability to meet those demands?	1.17	1.32
	P4: Thinking about the interpersonal demands of your job, how do you rate your current ability to meet those demands?	1.34	1.39
Job stress (1–4)	JS1: My job is physically demanding	2.43	0.99
	JS2: I am under constant time pressure due to a heavy workload	2.18	0.93
	JS3: I have very little freedom to decide how I do my work	1.88	0.81
	JS4: Considering the things I have to do at work, I have to work very fast	2.57	0.82
	JS5: I often feel bothered or upset in my work	1.92	0.74
	JS6: The demands of my job interfere with my personal life.	1.97	0.80
Co-worker support (1–4)	CS1: My co-workers listen to me when I need to talk about work-related problems.	3.18	0.63
	CS2: My co-workers help me with difficult tasks	3.13	0.67
	CS3: My co-workers help me in crisis situations at work	3.15	0.68
Supervisor support (1–4)	SS1: My supervisor is helpful to me in getting the job done.	3.03	0.70
	SS2: My supervisor is willing to extend himself/herself to help me perform my job.	2.98	0.81
	SS3: My supervisor takes pride in my accomplishments at work.	3.10	0.76
	SS4: My supervisor tries to make my job as interesting as possible.	2.86	0.81

### 2.3. Statistical Analysis

The chi-square test of goodness-of-fit was used to analyse the representativeness of study samples. SEM analysis was used to disentangle the complicated relationships between co-worker support, supervisor support, job stress, and presenteeism [46]. Data preparation and all statistical analyses were done with SPSS 21.0 (IBM Corp.: Armonk, NY, USA) and AMOS 21.0 (IBM Corp.: Armonk, NY, USA), unless otherwise stated. Missing values for observed indicators were imputed using expectation-maximization (EM) implementation of maximum likelihood [47,48]. There were no missing data on presenteeism, job stress, co-worker support, or supervisor support in the sample.

In SEM, four latent variables—presenteeism, job stress, co-worker support, and supervisor support—were first constructed using the indicators from PAWS, namely, the job stress scale, co-worker support scale, and supervisor support scale. Before imputing these indicators into SEM, Pearson correlation analysis was used to check whether the correlations between presenteeism, job stress, co-worker support, and supervisor support were significant [49].

Several recommendations for SEM have been made regarding evaluation methods and sample size for non-normally distributed data when the Normality Test does not support the normality assumption of measured variables. The criteria used to evaluate good global fit were a root mean square error of approximation (RMSEA) less than 0.08; goodness of fit index (GFI), normed fit index (NFI), comparative fit index (CFI), and Tucker–Lewis index (TLI) values of 0.90 or higher; and the smallest expected cross-validation index (ECVI), taking into account the large sample size [50]. Gold *et al.* [51] maintained that, when the sample is between 500 and 1000, EM implementation of maximum likelihood is much better than using the asymptotically distribution-free method in the model. The sample size in this study was 1649; thus, the method used to evaluate the model and sample size fulfilled both these criteria.

To determine if standardized regression coefficients *(*β*)* differed by subgroup, we conducted subgroup analyses of two age groups and two health status groups. To ensure that the two subgroups were of equal size, age was categorized as old (≥59 years; 58 was the median of the sample in this study) and young (50–58 years), based on the median (58 years) of the final sample. Health status was divided into two categories: above-average (excellent or very good health) and average-poor (good, fair or poor health).

## 3. Results

### 3.1. Demographic Characteristics of Participants

Among the 1649 participants, 54.5% were female and 45.5% were male. Most (67.6%) were married, 19.8% were separated or divorced, 6.7% were widowed, and 5.9% had never married. A quarter of respondents were white (26.7%), 9.9% were African American, 3.8% did not specify an ethnicity, and 59.6% had missing data on ethnicity. Mean age was 59.03 years (SD, 6.80), and the age range was 50 to 88 years. About 46.3% of respondents had finished high school, and 39.2% had a college degree. The mean number of working hours per week was 38.38 (SD, 12.35) and ranged from 1 to 95 h. Most respondents (64.9%) reported working more than 40 h per week. Most responders (75.7%) had at least one chronic disease. Hypertension (48.8%) and arthritis (41.4%) were most common, followed by diabetes mellitus (18.4%), heart disease (13.6%), and emotional/psychiatric problems (12.8%). Interestingly, most rated their health status as good or better. There was no significant difference in sex, education, marital status, health conditions, or chronic diseases between the initial participants and the final subset, except for age, race, and working hours (Table 1).

The means for the four presenteeism items (Table 2) were low and very similar, ranging from 1.17 (current ability to meet the mental demands of your job; SD = 1.32) to 1.38 (current ability to work; SD = 1.64). The means for the six job stress items were moderate and varied substantially, from 1.88 (very little freedom to decide how to do the work, SD = 0.81) to 2.57 (I have to work very fast, SD = 0.82). The means for the three co-worker support items were high and similar, ranging from 3.13 (my co-workers help me with difficult tasks, SD = 0.67) to 3.18 (my co-workers listen to me when I need to talk about work-related problems, SD = 0.63). The means for the four supervisor support items were also high but slightly lower than those for co-worker support, ranging from 2.86 (my supervisor tries to make my job as interesting as possible, SD = 0.81) to 3.10 (my supervisor takes pride in my accomplishments at work, SD = 0.76).

### 3.2. Pearson Correlations between Presenteeism, Job Stress, and Work Support

The correlation coefficients (r) for items within the same construct (Table 3) were strongly positively correlated (*r* = 0.60–0.76 for presenteeism, *r* = 0.14–0.46 for job stress, *r* = 0.69–0.82 for co-worker support, and *r* = 0.71–0.87 for supervisor support). Presenteeism was significantly positively correlated with all job stress items (*r* = 0.04–0.27) except item JS4. Presenteeism was significantly inversely correlated with co-worker support (*r* = −0.11 to −0.25) and supervisor support (*r* = −0.01 to −0.22). Job stress was significantly inversely correlated with co-worker support (*r* = −0.07 to −0.26) and supervisor support (*r* = −0.07 to −0.33).

**Table 3:** Intercorrelations between presenteeism (P), job stress, co-worker support (CS), and supervisor support (SS) items (** *p* < 0.01).

Items	P1	P2	P3	P4	JS1	JS2	JS3	JS4	JS5	JS6	CS1	CS2	CS3	SS1	SS2	SS3
P2	0.756 **															
P3	0.645 **	0.631 **														
P4	0.604 **	0.597 **	0.748 **													
JS1	0.159 **	0.269 **	0.148 **	0.140 **												
JS2	0.089 **	0.140 **	0.152 **	0.160 **	0.214 **											
JS3	0.166 **	0.184 **	0.201 **	0.213 **	0.180 **	0.404 **										
JS4	0.007	0.047	0.045	0.047	0.245 **	0.462 **	0.287 **									
JS5	0.157 **	0.146 **	0.237 **	0.260 **	0.139 **	0.414 **	0.376 **	0.299 **								
JS6	0.133 **	0.168 **	0.174 **	0.182 **	0.166 **	0.431 **	0.325 **	0.299 **	0.381 **							
CS1	−0.169 **	−0.178 **	−0.181 **	−0.252 **	−0.096 **	−0.148 **	−0.242 **	−0.078 **	−0.257 **	−0.234 **						
CS2	−0.118 **	−0.106 **	−0.132 **	−0.195 **	−0.075 **	−0.174 **	−0.216 **	−0.094 **	−0.238 **	−0.218 **	0.713 **					
CS3	−0.157 **	−0.124 **	−0.158 **	−0.207 **	−0.081 **	−0.136 **	−0.206 **	−0.070 **	−0.244 **	−0.189 **	0.686 **	0.816 **				
SS1	−0.126 **	−0.106 **	−0.153 **	−0.223 **	−0.084 **	−0.213 **	−0.268 **	−0.120 **	−0.315 **	−0.284 **	0.534 **	0.573 **	0.567 **			
SS2	−0.099 **	−0.086 **	−0.120 **	−0.199 **	−0.082 **	−0.228 **	−0.275 **	−0.135 **	−0.313 **	−0.288 **	0.517 **	0.540 **	0.521 **	0.866 **		
SS3	−0.134 **	−0.114 **	−0.141 **	−0.217 **	−0.081 **	−0.161 **	−0.277 **	−0.071 **	−0.325 **	−0.245 **	0.489 **	0.452 **	0.468 **	0.689 **	0.712 **	
SS4	−0.105 **	−0.110 **	−0.121 **	−0.170 **	−0.065 **	−0.202 **	−0.302 **	−0.128 **	−0.315 **	−0.283 **	0.472 **	0.451 **	0.449 **	0.709 **	0.737 **	0.746 **

### 3.3. Structural Equation Modelling

In the initial model, the path from supervisor support to presenteeism was insignificant (β = 0.03, *p* > 0.05). Hence, the path from supervisor support to presenteeism was eliminated. The criteria for fitness, such as REMSA, ECVI, GFI, CFI and TLI, indicated that the revised model was more appropriate.

**Figure 2:**
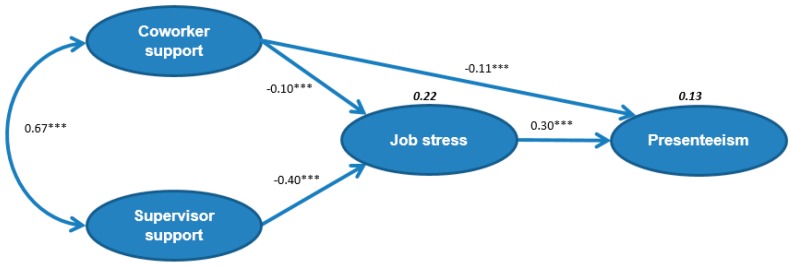
Final model of how co-worker and supervisor support affect job stress and presenteeism. (Numbers not in bold are the standardized regression coefficients and numbers in bold are explained variability, χ^2^/degrees of freedom = 4.840, root mean square error of approximation = 0.048, goodness normed fit index = 0.963, comparative fit index = 0.973, Tucker–Lewis index = 0.966, expected cross-validation index = 0.375; *** *p* < 0.001).

In the final model, job stress had a significant direct positive effect on presenteeism (β = 0.30; *p* < 0.001). Co-worker support had moderate direct negative effects on presenteeism (β = −0.11; *p* < 0.001) and job stress (β = −0.10; *p* < 0.001). Supervisor support had a direct negative effect on job stress (β = −0.40; *p* < 0.001). There was significant positive relationship between co-worker support and supervisor support (β = 0.67; *p* < 0.001). Co-worker support and supervisor support explained 22% of variability in job stress. Co-worker support, supervisor support, and job stress explained 13% of variability in presenteeism (Figure 2).

Subgroup analyses showed a different pattern regarding the effect of co-worker support on job stress and presenteeism. Among the younger participants and those with average-poor health, co-worker support did not significantly affect job stress but continued to significantly influence presenteeism. Among the participants with above-average health, co-worker support did not significantly affect job stress or presenteeism (Table 4).

**Table 4:** Standardized regression coefficients (β) with *p* values (α = 0.05) for the components of subgroup analyses.

Paths	Above−Average Health (*n* = 892)		Average−Poor Health (*n* = 757)		Young (50–58 Years, *n* = 893)		Old (59–80 Years, *n* = 756)	
	β	*p* value	β	*p* value	β	*p* value	β	*p* value
CS to JS	−0.06	0.327	−0.10	0.08	−0.08	0.147	−0.13	*
SS to JS	−0.43	***	−0.38	***	−0.41	***	−0.37	***
JS to presenteeism	0.28	***	0.29	***	0.28	***	0.37	***
CS to SS	0.69	***	0.62	***	0.64	***	0.70	***
CS to presenteeism	−0.03	0.424	−0.11	*	−0.18	*	−0.11	*

CS, co-worker support; SS, supervisor support; JS, job stress; * Significant at α = 0.05; *** significant at *p* < 0.001.

## 4. Discussion

Presenteeism was low in this representative sample of older US workers. Job stress was moderate, and co-worker and supervisor support were high. SEM revealed that job stress had a significant direct positive effect on presenteeism (β = 0.30). Co-worker support had significant direct negative effects on presenteeism (β = −0.11) and job stress (β = −0.10). Supervisor support had an insignificant effect on presenteeism but a significant negative effect on job stress (β = −0.40). Co-worker support and supervisor support were highly correlated (β = 0.67).

The path with the highest standardized maximum-likelihood estimation was the inter-relation between co-worker support and supervisor support (β = 0.67). Social capital theory [52,53,54,55,56,57] holds that both co-worker support from the horizontal dimension (that is, social contacts and level of trust in relation to co-workers) and supervisor support from the vertical dimension (that is, the relation with a supervisor across different levels of power dimensions) indispensably contribute to a supportive work environment by reducing job stress and strain [58,59]. This supportive work environment not only leads to trusting relations at work, thereby enabling employees to access resources—it was also associated with better health status in both individual-level [60] and multilevel [61] studies. This is consistent with our findings and the results of subgroup analyses. However, to our knowledge, the correlation between co-worker and supervisor support has not been carefully examined previously [62], although studies have separately studied the effects of co-worker and supervisor support in different industries [63,64]. Our findings indicate that aging US workers have good relationships with their colleagues and supervisors and therefore work in a more supportive work environment and have better health status, perhaps because of their long work experience in their positions. Thus, the importance and strong correlations of co-worker and supervisor support in the work environment with health status should be simultaneously examined in future studies of the aging workforce.

We investigated the buffering effects of work support on job stress and presenteeism and found that co-worker support significantly affects presenteeism and job stress and that supervisor support has a significant direct negative effect on job stress but not presenteeism. These findings accord with those of previous studies of co-worker support, supervisor support, and presenteeism [35,64,65,66]. The difference in the effects of co-worker and supervisor support on presenteeism could be due to the differing roles of co-workers and supervisors. The buffering model of social support holds that employees who lack social support shift their resources from current work tasks to managing high levels of job stress [67]. To cope with these job stresses, employees collaborate with co-workers in reducing their complaints and presenteeism. However, because resources for employees are always limited, and supervisors are the ones responsible for assigning work tasks to employees, it is reasonable to see a strong negative effect of supervisor support on job stress but only a modest negative effect of co-worker support on job stress.

Another reason for the difference in the effects of co-worker and supervisor support on presenteeism may be that the targets of work support differ. Whereas supervisor support more strongly affects job stresses such as workload [68], co-worker support has a stronger impact on job performance [63]. One logical interpretation of co-worker support is that employees who believe that they are supported by colleagues enjoy their work environment and thus excel in their work and perform better than those with less support. They feel comfortable requesting co-worker help in completing certain unclear tasks, which decreases presenteeism [69,70]. Our findings thus explain why supervisor involvement, especially supervisor support, is essential to the success of stress interventions in enterprises and why co-worker support should be the first priority when attempting to reduce presenteeism. Subgroup analysis showed that the negative effect of co-worker support on job stress was not significant among younger participants and that the negative effect of co-worker support on presenteeism was not significant among participants with above-average health. Studies of the social status of participants in the HRS indicate that more participants rated their social status as higher than middle class in the old group (74.9%) than young group (64.6%), and our results indicate that the main job stress for these older participants had become so organizationally broad (e.g., how to use limited resources to successfully manage an entire team or even the enterprise itself) that only experienced colleague could easily share these challenges, while these younger participants might have no such experienced colleague and still address their job stress relying on the support from their supervisors. Subsequently, from the perspective of the social capital theory [52,53,54,55,56,57], a supportive work environment consisting of good co-worker support and supervisor support enables employees with better health status to have less potential productivity loss due to health conditions, while employees with average-poor health status might choose sickness presenteeism.

The present findings confirm that job stress has a significant direct positive effect on presenteeism (β = 0.30), which is consistent with previous findings. An earlier study [71] reported a moderate relationship between presenteeism and job stress (Spearman correlation coefficient = 0.353–0.431, estimate with presenteeism) in analyses of a general population survey. That study assessed job stress from the dimensions of interpersonal relations, roles of stress, and intrinsic factors in work. In the present study, we viewed job stress from six dimensions (e.g., physical demands) in a comprehensive SEM model focusing on a representative aging workforce. The resulting findings are likely to be more accurate and specific to aging workers than those of previous studies.

We used SEM to construct a comprehensive model that examined the relationships between presenteeism, job stress, co-worker support, and supervisor support, while considering the job demand–resources and buffering model of work support. In our case, only 22% of the variability in job stress was explained by co-worker and supervisor support, and 13% of the variability of presenteeism was explained by job stress, co-worker support, and supervisor support. Low variability percentages are common when the outcome variables are perceptions, attitudes, and behaviours [72]. Attitude and behaviour are very subjective and vary a great deal inter- and intra-personally; thus, our model is still robust. Future studies are likely to use this model to investigate different populations.

We believe that presenteeism in an aging workforce has unique characteristics that have not been directly studied previously. The present level of presenteeism was low, job stress was moderate, and co-worker and supervisor support were high. These results indicate that a high level of work support could effectively address the negative effects of job stress and their impact on presenteeism. Previous studies [73,74] reported that presenteeism was lower among older adults than among younger adults, but this phenomenon has not been directly studied. There are three possible explanations for the lower presenteeism among older adults. Firstly, older workers may grow accustomed to job stress during the previous 30 to 40 years of work. Job stress might no longer affect their work performance to the extent that it affects younger workers. Secondly, as more than 70% of participants rated their social status as higher than middle class, it indicates that most of the aging workers were supervisors. Thus, as compared with participants who had supervisor-related job stress, they might have had fewer work difficulties, which could lower job stress and presenteeism. Thirdly, aging participants might have stronger social support and responsibility for their work because of their long work experience [75]. Policymakers should be made aware of the importance of the aging workforce. Because of their reliability and professionalism, aging workers have a key role in addressing the challenges of an aging society.

Therefore, among the aging working population, we suggest that supervisors share difficult work tasks among employees and build a supportive work culture to reduce presenteeism, increase employee job satisfaction [76] and organizational commitment [77], and decrease turnover [78]. Additionally, reciprocation of work support between lower-level employees and managers may decrease job stress and increase productivity [79]. Combined co-worker and supervisor support is effective in reducing job stress and improving presenteeism among an aging working population. Ultimately, interventions targeting job stress—such as flexible work scheduling, workload sharing among supervisors, co-workers, and employees, and appropriate supervisor behaviour and attitudes toward employees—can substantially reduce stress and presenteeism.

## 5. Limitations

Several limitations warrant attention. Firstly, our results may not be valid for young working populations and other countries because our population was mainly older US employees. Nevertheless, our findings will be helpful to policymakers from countries and regions facing the challenges of an aging society, especially those in Europe, Japan, and China. Secondly, the use of secondary data in the present study limited the selection of target variables for our model. For example, workplace policies are an important issue in presenteeism but were not included in the HRS data. This is a common criticism of social science research, as it nearly impossible to consider all relevant constructs within a model. Thirdly, our use of self-reported presenteeism rather than objective measures may limit the generalizability of our findings. Future research should analyse both subjective and objective data. Fourthly, to differentiate supervisor support and co-worker support at the workplace, we did not consider other aspects of social support in this study, such as work–life enhancement and interference. Fifth, we chose to use a cross-sectional study design because data for some of the investigated variables were only available in 2010. Future research should use longitudinal designs to investigate the relations between co-worker support, supervisor support, job stress, and presenteeism. Finally, we did not consider both the positive and negative aspects of presenteeism and job stress in this study. This also limits the generalizability of our model and conclusions.

## 6. Conclusions

We used a comprehensive framework to analyze data from a representative national survey of older US workers. Co-worker support had a direct negative effect on job stress and presenteeism, and supervisor support had a direct negative effect on job stress but not presenteeism. Presenteeism was lower among the elder participants. The results suggest that presenteeism is reduced by increased attention to employee stress at the workplace, by greater support at work from colleagues and employers, and by comfortable interpersonal relationships among colleagues and between employers and employees.

## References

[1] Simpson R. (1998). Presenteeism, power and organizational change: Long hours as a career barrier and the impact on the working lives of women managers. Br. J. Manag..

[2] Worrall L., Cooper C., Campbell F. (2000). The new reality for UK managers: Perpetual change and employment instability. Work Employ. Soc..

[3] Virtanen M., Kivimaki M., Elovainio J., Vahtera J., Ferrie J.E. (2003). From insecure to secure employment: Changes in work, health, health related behaviours, and sickness absence. Occup. Environ. Med..

[4] Koopman C., Pelletier K.R., Murray J.F., Sharda C.E., Berger M.L., Turpin R.S., Hackleman P., Gibson P., Holmes D.M., Bendel T. (2002). Stanford Presenteeism. Scale: Health status and employee productivity. J. Occup. Environ. Med..

[5] Burton W.N., Morrison A., Wertheimer A.I. (2003). Pharmaceuticals and worker productivity loss: A critical review of the literature. J. Occup. Environ. Med..

[6] Johnson S.K. (2008). Medically Unexplained Illness: Gender and Biopsychosocial Implications.

[7] Johns G. (1997). Contemporary research on absence from work: Correlates, causes, and consequences. Int. Rev. Ind. Organ. Psychol..

[8] Johns G., Anderson N., Ones D.S., Sinangil H.K., Viswesvaran C. (2001). The psychology of lateness, absenteeism, and turnover. Handbook of Industrial, Work & Organizational Psychology.

[9] Johns G., Thomas J.C., Hersen M. (2002). Absenteeism and mental health. Handbook of Mental Health in the Workplace.

[10] Johns G. (2003). How methodological diversity has improved our understanding of absenteeism from work. Hum. Resour. Manag. Rev..

[11] Johns G., Cooper C.L., Barling J. (2008). Absenteeism and presenteeism: Not at work or not working well. The Sage Handbook of Organizational Behaviour.

[12] Aronsson G., Gustafsson K., Dallner M. (2000). Sick but yet at work. An. empirical study of sickness presenteeism. J. Epidemiol. Community Health.

[13] Dewa C.S., Lesage A., Goering P., Craveen M. (2004). Nature and Prevalence of Mental Illness in the Workplace. Healthc. Pap..

[14] Goetzel R.Z., Long S.R., Ozminkowski R.J., Hawkins K., Wang S., Lynch W. (2004). Health, absence, disability, and presenteeism cost estimates of certain physical and mental health conditions affecting U.S. employers. J. Occup. Environ. Med..

[15] Johns G. (2010). Presenteeism in the Workplace: A review and research agenda. J. Organ. Behav..

[16] The World Bank Life Expectancy From Birth Chart. http://data.worldbank.org/indicator/SP.DYN.LE00.IN.

[17] Centers for Disease Control and Prevention (1999). Ten great public health achievements—United States, 1900–1999. Morbidity and Mortality Weekly Report.

[18] Ilmarinen J., Tuomi K., Seitsamo J. (2005). New dimensions of work ability. Int. Congr. Ser..

[19] Ilmarinen J. (2005). Toward a Longer Worklife: Ageing and the Quality of Worklife in the European Union.

[20] Sundquist J., Ostergren P.O., Sundquist K., Johansson S.E. (2003). Psychosocial Working Conditions and Self-Reported Long-Term Illness: A Population-Based Study of Swedish-Born and Foreign-Born Employed Persons. Ethn. Health.

[21] Elstad J.I., Vabø M. (2008). Job stress, sickness absence and sickness presenteeism in Nordic elderly care. Scand. J. Public Health.

[22] Ryu I., Jeong D., Kim I., Roh J., Won J. (2012). Association Between Job Stress, Psychosocial Well-Being and Presenteeism, Absenteeism: Focusing on Railroad Workers. Korean J. Occup. Environ. Med..

[23] Kim J., Park S., Kim D., Kim H., Leem J., Lee E., Lee D., Lee J. (2009). Absence and Early Leave Status due to Job Stress and its Relationship to Job Stress Factors According to the Korean Occupational Stress Scale among Workers in Small and Medium Scale Industry. Korean J. Occup. Environ. Med..

[24] lazarus R.S. (1991). Pschological Stress and the Coping Process.

[25] Edwards J.R. (1992). A Cybernetic Theory of Stress, Coping, and Well-Being in Organizations. Acad. Manag. Rev..

[26] Hobfoll S.E. (2001). The Influence of culture, community, and the nested-self in the stress process: Advancing conservation of resources theory. Appl. Psychol..

[27] Sloan M.M. (2012). Unfair Treatment in the Workplace and Worker Well-Being: The Role of Co-worker Support in a Service Work Environment. Work Occup..

[28] Edwards J.R., Rothbard N.P. (1999). Work and family stress and well-being: An. examination of person–environment fit in the work and family domains. Organ. Behav. Hum. Decis. Process..

[29] Pritchard R.D., Karasick B.W. (1973). The Effects of Organizational Climate on Managerial Job Performance and Job Satisfaction. Organ. Behav. Hum. Decis. Process..

[30] Cooper C.L., Dewe P., O’Driscoll M.P. (2001). Organizational Stress: A Review and Critique of Theory, Research, and Applications.

[31] Otsuka Y., Takahashi M., Nakata A., Haratani T., Kaida K., Fukasawa K., Hanada T., Ito A. (2007). Sickness absence in relation to psychosocial work factors among daytime workers in an electric equipment manufacturing company. Ind. Health.

[32] Boz M., Martínez-Corts I., Munduate L. (2009). Breaking negative consequences of relationship conflicts at work: The moderating role of work family enrichment and supervisor support. Rev. Psicol. Trab. Organ..

[33] Gouldner A.W. (1960). The norm of reciprocity: A preliminary statement. Am. Sociol. Rev..

[34] Cummins R.C. (1990). Job Stress and the Buffering Effect of Supervisory Support. Group Organ. Manag..

[35] Mayo M., Sanchez J.I., Pastor J.C., Rodriguez A. (2012). Supervisor and co-worker support: A source congruence approach to buffering role conflict and physical stressors. Int. J. Hum. Resour. Manag..

[36] Kristof-Brown A.L., Zimmerman R.D., Johnson E.C. (2005). Consequences of individuals’ fit at work: A meta-analysis of person-job, person-organization, person-group, and person-supervisor fit. Pers. Psychol..

[37] U.S. Department of Health and Human Services (2007). Growing Older in America: The Health and Retirement Study.

[38] Health and Retirement Study Produced and Distributed by the University of Michigan with Funding from the National Institute on Aging (Grant Number NIA U01AG009740).

[39] Smith J., Fisher G., Ryan L., Clarke P., House J., Weir D. (2013). Psychosocial and Lifestyle Questionnaire 2006–2010 Documentation Report Core Section LB.

[40] Juster F.T., Suzman R. (1995). An overview of the Health and Retirement Study. J. Hum. Resour..

[41] Vänni K., Virtanen P., Luukkaala T., Nygård C.H. (2012). Relationship between perceived work ability and productivity loss. Int. J. Occup. Saf. Ergon..

[42] Ilmarinen J., Rantanen J. (1999). Promotion of Work Ability During Ageing. Am. J. Ind. Med..

[43] Quinn R.P., Staines G.L. (1984). The 1977 Quality of Employment Survey.

[44] Haynes C.E., Wall T.D., Bolden R.I., Stride C., Rick J.E. (1999). Measures of perceived work characteristics for health services research: Test of a measurement model and normative data. Br. J. Health Psychol..

[45] Eisenberger R., Stinglhamber F., Vandenberghe C., Sucharski I.L., Rhoades L. (2002). Perceived supervisor support: Contributions to perceived organizational support and employee retention. J. Appl. Psychol..

[46] Van der Kline R.B. (2005). Principles and Practice of Structural Equation Modelin.

[47] Rubin D.B. (1976). Inference and missing data. Biometrika.

[48] Royston P. (2004). Multiple imputation of missing values. Stata J..

[49] Cohen J., Cohen P.C., West S.G., Aiken L.S. (2003). Applied Multiple Regression/Correlation Analysis for the Behavioral Sciences.

[50] Ullman J. (1996). Structural equation modelling. Using Multivariate Statistics.

[51] Gold M.S., Bentler P.M., Kim K.H. (2002). A Comparison of Maximum-Likelihood and Asymptotically Distribution-Free Methods of Treating Incomplete Non-Normal Data. Struct. Equ. Model..

[52] Seibert S.E., Kraimer M.L., Liden R.C. (2001). A Social Capital Theory of Career Success. Acad. Manag. J..

[53] Kouvonen A., Oksanen T., Vahtera J. (2008). Low workplace social capital as a predictor of depression: The Finnish Public Sector Study. Am. J. Epidemiol..

[54] Putnam R. (2001). Bowling Alone: The Collapse and Revival of American Community.

[55] McKenzie K., Whitley R., Weich S. (2002). Social capital and mental health. Br. J. Psychiatry.

[56] Almedom A.M. (2005). Social capital and mental health: An interdisciplinary review of primary evidence. Soc. Sci. Med..

[57] Islam M.K., Merlo J., Kawachi I., Lindström M., Gerdtham U.-G. (2006). Social capital and health: Does egalitarianism matter? A literature review. Int. J. Equity Health.

[58] Wiskow C., Albreht T., de Pietro C. (2010). How to create an attractive and supportive working environment for health professionals. Health Systems and Policy Analysis.

[59] European Agency for Occupational Safety and Health (2009). OSH in Figures: Stress at Work—Facts and Figures.

[60] Mohseni M., Lindstrom M. (2007). Social capital, trust in the health care system and self-rated health: The role of access to health care in a population-based study. Soc. Sci. Med..

[61] Kawachi I., Kennedy B.P., Lochner K. (1997). Long live community: Social capital as public health. Am. Prospect.

[62] Stamper C.L., Johlke M.C. (2003). The Impact of Perceived Organizational Support. on the Relationship between Boundary Spanner Role Stress and Work Outcomes. J. Manag..

[63] Schaubroeck J., Cotton J., Jennings K. (1988). Antecedents and Consequences of Role Stress: A Covariance Structure Analysis. J. Organ. Behav..

[64] AbuAlRub R.F. (2004). Job Stress, Job Performance, and Social Support. Among Hospital Nurses. J. Nurs. Scholarsh..

[65] Jourdain G., Vézina M. (2013). How psychological stress in the workplace influences presenteeism propensity: A test of the Demand–Control–Support model. Eur. J. Work Organ. Psychol..

[66] Lu L., Cooper C.L., Lin H.Y. (2013). A cross-cultural examination of presenteeism and supervisory support. Career Dev. Int..

[67] Halbesleben J.R.B., Bowler W.M. (2007). Emotional exhaustion and job performance: The mediating role of motivation. J. Appl. Psychol..

[68] Kirmeyer S.L., Dougherty T.W. (1988). Work load, tension, and coping: Moderating effects of supervisor support. Pers. Psychol..

[69] Mitchell M.S., Ambrose M.L. (2007). Abusive supervision and workplace deviance and the moderating effects of negative reciprocity beliefs. J. Appl. Psychol..

[70] Hoobler J.M., Brass D.J. (2006). Abusive supervision and family undermining as displaced aggression. J. Appl. Psychol..

[71] Umann J., de Azevedo Guido L., da Silva R.M. (2014). Stress, coping and presenteeism in nurses assisting critical and potentially critical patients. Rev. Esc. Enferm. USP.

[72] Armitage C.J., Conner M. (2001). Efficacy of the Theory of Planned Behaviour: A meta-analytic review. Br. J. Soc. Psychol..

[73] Hansen C.D., Andersen J.H. (2008). Going ill to work: What personal circumstances, attitudes and work-related factors are associated with sickness presenteeism?. Soc. Sci. Med..

[74] Jeon S.H., Leem J.H., Park S.G., Heo Y.S., Lee B.J., Moon S.H., Jung D.Y., Kim H.C. (2014). Association among Working Hours, Occupational Stress, and Presenteeism. among Wage Workers: Results from the Second Korean Working Conditions Survey. Ann. Occup. Environ. Med..

[75] Silverstein M. (2008). Meeting the challenges of an aging workforce. Am. J. Ind. Med..

[76] Hamaideh S.H. (2011). Burnout, Social Support, Job Satisfaction among Jordanian Mental Health Nurses. Issues Ment. Health Nurs..

[77] Robert E., Peter F., Valerie D.-L. (1990). Perceived organizational support and employee diligence, commitment, and innovation. J. Appl. Psychol..

[78] Kim H., Stoner M. (2008). Burnout and Turnover Intention Among Social Workers: Effects of Role Stress, Job Autonomy and Social Support. Adm. Soc. Work.

[79] Eisenberger R., Armeli S., Rexwinkel B., Lynch P., Rhoades L. (2001). Reciprocation of perceived organizational support. J. Appl. Psychol..

